# Can Natural Products Exert Neuroprotection without Crossing the Blood–Brain Barrier?

**DOI:** 10.3390/ijms22073356

**Published:** 2021-03-25

**Authors:** Manon Leclerc, Stéphanie Dudonné, Frédéric Calon

**Affiliations:** 1 Faculté de Pharmacie, Université Laval, Québec, QC G1V 0A6, Canada; manon.leclerc.4@ulaval.ca; 2 Axe Neurosciences, Centre de Recherche du CHU de Québec–Université Laval, Québec, QC G1V 4G2, Canada; 3 Institut sur la Nutrition et les Aliments Fonctionnels (INAF), Québec, QC G1V 0A6, Canada; stephanie.dudonne.1@ulaval.ca; 4 OptiNutriBrain-Laboratoire International Associé (NutriNeuro France-INAF Canada), Québec, QC G1V 0A6, Canada

**Keywords:** blood–brain barrier, central nervous system, bioavailability, polyphenols, omega-3 polyunsaturated fatty acids, gut–brain axis

## Abstract

The scope of evidence on the neuroprotective impact of natural products has been greatly extended in recent years. However, a key question that remains to be answered is whether natural products act directly on targets located in the central nervous system (CNS), or whether they act indirectly through other mechanisms in the periphery. While molecules utilized for brain diseases are typically bestowed with a capacity to cross the blood–brain barrier, it has been recently uncovered that peripheral metabolism impacts brain functions, including cognition. The gut–microbiota–brain axis is receiving increasing attention as another indirect pathway for orally administered compounds to act on the CNS. In this review, we will briefly explore these possibilities focusing on two classes of natural products: omega-3 polyunsaturated fatty acids (n-3 PUFAs) from marine sources and polyphenols from plants. The former will be used as an example of a natural product with relatively high brain bioavailability but with tightly regulated transport and metabolism, and the latter as an example of natural compounds with low brain bioavailability, yet with a growing amount of preclinical and clinical evidence of efficacy. In conclusion, it is proposed that bioavailability data should be sought early in the development of natural products to help identifying relevant mechanisms and potential impact on prevalent CNS disorders, such as Alzheimer’s disease.

## 1. Introduction

When it comes to clinical efficiency, natural products are not very different from synthetic drugs. It is generally agreed that the ultimate clinical efficiency of a drug generally depends on three main factors. The first is efficacy, which usually attracts the most the attention of researchers. In a nutshell, this can be summarized as whether a drug interacts with sufficient affinity with its receptor to induce a dose-dependent pharmacological effect. The second is safety, which can only be fully determined in clinical phases after a wide usage. It can be predicted in preclinical phases, but bad surprises are not the exception, leading to rejection of drugs at late clinical stages. The third, and probably most often neglected factor, is bioavailability, which by definition must be quantitative. These factors are equality important for natural products as they are for synthetic drugs.

Bioavailability can be summarized as the actual concentration of the drug at the target site, after taking into account ADME and PK (absorption, distribution, metabolism, excretion, toxicology and pharmacokinetics), in relation to time. Bioavailability is quantitative, and its determination thus requires adequate analytic capabilities [1]. For medical indications involving the central nervous system (CNS), the blood–brain barrier (BBB) stands as an additional barrier to be crossed. For most drugs, a low bioavailability within the brain is more the rule than the exception [2,3]. Research on disease-modifying treatments for CNS diseases have generated a cemetery of failed drugs, rejected in part because of their incapacity to cross the BBB [3,4,5,6,7]. Thus, it is crucial that the biodistribution of a natural or a synthetic product is known early in its development in order to anticipate its therapeutic efficacy and limit adverse effects, particularly when a bioactivity in the CNS is considered essential.

In the face of the bulk of evidence accumulated over the years, it is becoming difficult to ignore that natural products have an impact on brain function [8,9]. However, their use in disease conditions in a real clinical context, combined or not with currently approved interventions, remains to be clarified. Besides legal considerations [10], poor understanding of their exact mechanisms of action remain an obstacle to full development. Still, from a pharmacoeconomic standpoint, it is obvious that benefit/(risk+cost) ratio of natural products are often very high, compared to patented high-cost biopharmaceuticals available, and thus deserve intense research efforts.

## 2. Omega-3 Polyunsaturated Fatty Acids: Effect on Cognition

There is a convincing volume of epidemiological studies showing associations between high omega-3 polyunsaturated fatty acids (n-3 PUFAs) consumption from marine sources, high docosahexaenoic acid (DHA) or eicosapentaenoic acid (EPA) blood levels and lower risk of dementia/Alzheimer’s disease (AD) or better cognitive function [11,12,13,14,15,16]. Results from randomized controlled trials (RCTs) are more mitigated. While negative in patients already diagnosed with dementia, the frequency of positive RCTs increases when volunteers are recruited before AD diagnosis [13,14,17,18,19,20,21]. As with many other natural products, confounding variables such as dietary intake, genetic background and metabolism hinder the generalization of findings [17,22].

A strong trend noted in the AD field in the last decade is an increased recognition of the importance of modifiable risk factors, such as nutrition. Omega-3 PUFAs and other dietary factors are now a key part of most multidomain interventions aimed at preventing dementia. Three such studies have been published so far showing no effect on the incidence of dementia but significant benefit on cognitive tests [14,23,24,25]. Interestingly, in the Multidomain Alzheimer Preventive Trial (MAPT) study, the inclusion of a higher n-3 PUFA intake in the intervention appeared to have an additive effect on Mini-Mental State Examination (MMSE) and Cardiovascular Risk Factors, Aging, and Incidence of Dementia (CAIDE) scores [23,26,27]. These multidomain intervention trials are the focus of large investments worldwide at the moment [25].

A key unanswered question is whether n-3 PUFAs act on the progression of cognitive deficits at the molecular level. Disease modification in AD and other neurodegenerative diseases is very difficult to demonstrate in clinics, due to challenges in study design, but also due to the lack of reliable biomarkers [28,29,30]. Structural MRI provides a way to assess the volume of specific brain regions highly involved in cognitive performance. An increasing number of MRI-based studies provide some evidence of an association between fish intake and favorable changes in brain integrity [31,32].

Animal studies display a strong potential to provide additional insights on such questions. There are in fact many reports on the effect of n-3 PUFA supplementation on β-amyloid (Aβ) [33] and synaptic neuropathologies [34], but less on the accumulation of tau (reviewed in [18,35,36]). Omega-3 PUFAs may also act more directly on neuronal function by progressively integrating cell membranes, without necessarily targeting AD neuropathology per se [37,38,39,40]. In animal models of the nigrostriatal denervation observed in Parkinson’s disease (PD), not only neuroprotective [41,42] but also neurorestorative [43] actions of DHA have been reported, with more limited effect on synucleinopathy [44]. It should be noted that animal studies are not without conflicting results and may involve some level of publication bias, given the difficulty of publishing results perceived as negative, which may blur the picture. Nevertheless, data accumulated so far provide arguments to consider that n-3 PUFAs may exert disease modification.

## 3. Omega-3 Polyunsaturated Fatty Acids: Confirmed CNS Bioavailability

The capacity of dietary n-3 PUFAs to reach the brain has been demonstrated decades ago, initially with deprivation studies, and then with supplementation studies, later replicated numerous times [18,34,39,45,46,47,48,49,50,51,52,53]. Dietary investigations in animals consistently show that DHA intake leads to a corresponding accretion of DHA in cerebral tissue, with limited interindividual variability [33,34,37,42,43,44,45,54,55,56,57,58,59]. Additional studies have shown that DHA, EPA or arachidonic acid (ARA) can cross the BBB through a nonsaturable uptake mechanism [35,60,61,62,63]. This comes as no surprise to the eyes of a neuropharmacologist, as the chemical structure of fatty acids (FAs) suggests free diffusion across lipid membranes forming the BBB [35,48,64]. A relatively small molecular size, very few potential hydrogen bonds and highly lipophilic moieties are all key characteristics of brain-penetrant molecules [65,66]. Additional studies in animal models have confirmed the importance of diffusion of plasma nonesterified DHA to supply the brain [63]. Still, an ability to cross the BBB does not equate optimal bioavailability in the brain. Relatively few DHA molecules are found as a free unbound form in the blood. Most DHA is incorporated in several more stable circulating complexes, which may increase the area under the curve (AUC) and the amount ultimately bioavailable for the brain [63,67,68].

The data summarized here form a solid basis to conclude that a proportion of n-3 PUFAs ingested in food or supplement will end up in the brain. However, these proportions can be modulated, through BBB transport, peripheral metabolism and pathological status. For instance, brain transport [69] and bioavailability [70] are decreased by the expression of apolipoprotein ε4 (ApoE4) [51]. Brain uptake is also modulated by AD transgene expression in the mouse brain [22,71]. Importantly, the levels of PUFAs in the CNS need to be maintained. Uptake is just one of the many variables that ultimately determine CNS concentrations of each fatty acid. Proteins like ACSL6, a member of the long-chain acyl-CoA synthetase family, or FABP5, a fatty-acid-binding protein, have been shown as essential for maintaining brain DHA levels [72,73,74]. In summary, there is overwhelming evidence that dietary intake of a specific FA, such as DHA, can lead to brain concentrations sufficient to interact with therapeutic targets in the brain. These concentrations, however, are susceptible to variations due to pathological anomalies, genetic background and ADME-related variability. It should be kept in mind that these factors may significantly impair n-3 PUFA bioavailability and lead to unpredictability of the therapeutic response in a clinical setting.

## 4. Polyphenols: Brain Health

Polyphenolic compounds are phytochemicals generally classified as flavonoids, including flavonols, flavan-3-ols, flavones, flavanones, isoflavones and anthocyanins; and nonflavonoids such as phenolic acids, hydroxycinnamic acids, lignans, stilbenes and tannins [75]. Despite a large heterogeneity of data reported in literature, the mean total polyphenol intake has been estimated at around 1g per day, the highest intake being commonly associated with the now intensively studied Mediterranean-like diets [76,77,78]. The list of studies on the potential neuroprotective effects of various polyphenols is very long, and they have been the subject of several comprehensive reviews [9,79,80]. Overall, there is a compelling amount of epidemiological, clinical and preclinical evidence that selected polyphenols could improve cognitive performance and be considered in a preventive setting against age-related cognitive loss and neurodegenerative diseases [9,79,81,82,83]. The most compelling evidence documented so far are for coffee, cocoa and tea, the most common sources of polyphenols, mainly flavonoids [14,16,81,84,85,86,87]. In addition, berries, such as blueberry and grape, have shown potential to prevent neurodegeneration and cognitive decline [82,83,88,89,90,91,92]. Recent studies emphasize the association between higher dietary flavonoid intake and a lower incidence of AD dementia [93,94]. In contrast, a recent meta-analysis investigating a series of health endpoints found no significant association between polyphenol intake and cognitive ratings, such as the AD Assessment Scale–Cognitive Subscale (ADAS-Cog) [95]. As always, data from associative studies do not readily transfer into intervention studies and many clinical trials do not report any beneficial outcome, even with resveratrol [17,75,96]. For example, clinical trials with standardized ginkgo biloba extracts, which are rich in flavonoids, or curcumin formulations have led to disappointing results in early AD [19,97,98,99]. Epigallocatechin gallate (EGCG) is a flavan-3-ol extracted from green tea leaves that is being investigated in multiple-system atrophy, AD and other diseases [25,75,100,101]. Although several mechanisms have been proposed, EGCG inhibits the formation of toxic oligomers in vitro and may prevent the aggregation of amyloidogenic proteins [101,102,103,104,105]. Clinical trials in AD are still ongoing, but a phase III trial revealed no efficacy in multiple-system atrophy patients, and led to liver damage in some participants [101,106]. More positive results were reported in smaller RCTs focusing on berry extracts, in individuals with mild cognitive complaint [90,107].

As pointed out in many of the above-mentioned studies, discrepancies in associative studies and failure in clinical trials could be due to the low bioavailability of polyphenols. Most studies were reported without fully assessing the bioavailability and the chemistry of polyphenols. Another limitation is the wide variation in response to polyphenols, due to the interindividual variability of gut-microbiota composition. Indeed, a recent study showed, for example, that a subgroup of volunteers experiencing a higher rate of age-related cognitive decline also displayed a higher excretion rate of phenolic metabolites, suggesting that some individuals are less likely to benefit from polyphenols consumption [90]. Obviously, since polyphenols form a large family that includes over 8000 chemical structures [108], it has to be anticipated that clinical trials will lead to different responses. A key unescapable fact to note is that high intakes of berries and vegetables are not fully dissociable from lifestyle patterns. Nevertheless, the evidence gathered so far is strong enough that diets enriched in polyphenolic compounds are incorporated into ongoing multidomain lifestyle intervention trials launched to prevent dementia [25].

## 5. Polyphenols: Low Bioavailability

The bioavailability of the most common dietary polyphenols has been addressed in several reports [75,109,110,111,112,113,114,115,116,117,118]. Many of these cited reviews provide large tables comparing bioavailability data of series of selected polyphenols. They reveal that interaction with the food matrix, stability in the gastrointestinal tract, metabolic processes occurring in the intestine and the liver (phase I and II metabolism) and bacterial biodegradation mediated by gut microbiota are all key factors that lessen plasma bioavailability of most polyphenolic compounds [75,110,113,115,116].

The diversity of potentially circulating phenolic compounds is greatly amplified by the fact that they undergo massive metabolism after oral intake, leading to the generation of arrays of metabolites [108,114,115]. The chemical structure of phenolic compounds defines whether they are absorbed in the small intestine or reach the colon to be subjected to microbial catabolism. Substrates may be absorbed in the gut, appearing in plasma untransformed or as methylated, sulfated and glucuronidated derivatives following intestinal and hepatic phase II metabolism [108,115]. Such metabolic transformations occurring in the gut or the liver generally render molecules more hydrophilic, and thereby less likely to reach the CNS. Thus, the presence of phenolic metabolites in plasma or tissues is not necessarily a proof of bioavailability and bioactivity. Unabsorbed compounds (such as polymeric structures), along with phenolic metabolites released in the intestine through the enterohepatic recirculation, reach the colon where they are catabolized by the gut-resident microbes. Their chemical structure is considerably altered by the wide enzymatic repertoire of the intestinal bacteria, involving ring-fission and cleavage reactions of functional groups [119]. The generated microbial metabolites are absorbed from the colon and subjected to liver metabolism, resulting in circulating conjugated derivatives. This intensive microbial metabolism ultimately reduces the structural diversity of phenolic compounds to a limited number of low-molecular-weight metabolites. Unabsorbed polyphenols have been estimated at 90–95% of the ingested dose [120]. Bioactivities of phenolic compounds have therefore been mostly attributed to their microbial metabolites, detected in plasma of volunteers at concentrations similar to those shown to be effective in in vitro studies [108]. The gut microbiota is thus a key factor in regulating bioavailability of polyphenols and modulating their biological activities [108,114,115,121]. Whether polyphenols act per se or through their metabolites must be carefully considered in any efficacy studies.

To address this bioavailability issue, new formulations have been developed involving encapsulation or complexation of phenolic bioproducts [75,99,113,122]. For example, complexing blueberry and grape polyphenols with a plant-based protein blend has led to a significant improved stability in an in vitro gastrointestinal model [123]. Synergies between phytochemicals to improve their bioavailability have also been suggested and could be exploited. For example, enhancement of plasma concentrations of phenolic compounds from blueberries has been achieved with concomitant ingestion of flavan-3-ol-rich grape extract in rodents [124]. Similarly, conjugated metabolites of polyphenols from a strawberry−cranberry blend were found in higher concentrations in the plasma following a coingestion with a quercetin-rich onion extract in mice [125]. Additionally, combining polyphenols and probiotics can enhance bioavailability. An animal study previously reported such a synergy, in which plasma concentrations of cranberry phenolic microbial metabolites were significantly increased with a cotreatment with *Bacillus subtilis* [126]. Formulation of such synbiotics is currently being highlighted as a promising strategy to manage CNS disorders [127]. However, while such improved formulations may ameliorate intestinal bioaccessibility and plasma bioavailability of polyphenols, whether they translate to improved brain bioavailability is not established yet. Nevertheless, polyphenols are normally ingested with other food nutrients, so their bioavailability has to be interpreted globally [80,124,128].

Owing to the increased sensitivity of instrumentation [129,130], a rising number of studies have sought to measure polyphenols and their metabolites in the brains of rodents after systemic administration [75,112]. It is important to note that the determination of brain bioavailability requires proper methodologies and correct data interpretation. Studies inferring brain penetration are unfortunately not always well designed for that purpose. For example, many studies use brain samples still containing blood contamination, which obviously may confound any estimation of actual concentration in the brain parenchyma [1,131,132,133,134]. More advanced in vivo techniques to quantify transport through the BBB, such as in situ brain cerebral perfusion, are rarely utilized [135,136,137,138,139]. Finally, cerebrospinal fluid (CSF) levels are still used as a surrogate marker of brain penetration, while it has been known for a long time that many compounds transit from the blood to the CSF without entering the brain per se [3,131,140]. Although various quantitative methods clearly show the CNS bioavailability of n-3 PUFAs [18,22,35,51,57,60,61,62,63,67,68,71,74], very few have been applied to polyphenols [75,109,112,113]. In addition, reports on polyphenol CNS bioavailability vary enormously in terms of models, methods of measurement, doses, routes administration, incorporation within diets, extracts or lack thereof, correction for residual blood, type of data generated (qualitative or quantitative), units, formulation and excipients, etc. Such variability has been previously highlighted [75,112,113].

With these caveats in mind, brain concentrations ranging from pM to low nM concentrations are typically reported after high-dose administration of polyphenols, including with flavonoids [109,113,116,132,141,142,143]. Whereas the capability of some polyphenolic compounds to cross the BBB, such as sulfated and methylated phenolic acids, is supported by some reports, there is still a global lack of information regarding the biodistribution of phenolic microbial metabolites [109,113,144]. While we have a fairly good view of how n-3 PUFAs can cross the BBB, including their rate of transport [18,22,35,51,57,60,61,62,63,67,68,71,74], the exact mechanisms that could mediate the uptake of polyphenols into the brain remain elusive [75,109,110,112,113]. In sum, although this remains controversial, most well-designed studies show that most ingested phytochemicals can be found at best at very low levels at therapeutic sites in the brain, likely under the minimum effective concentration (MEC).

## 6. How Can Polyphenols Act on the Brain?

**Figure 1:**
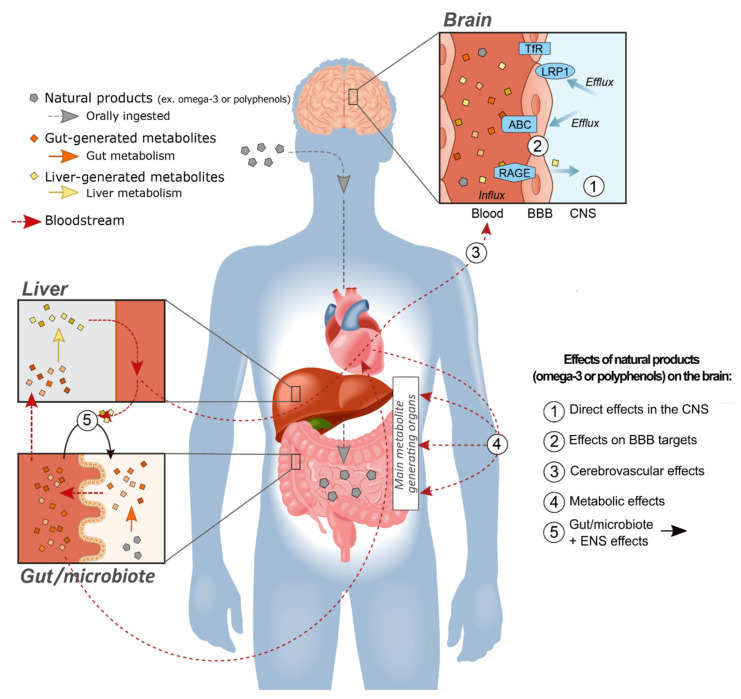
Natural products (NPs), such as omega-3 polyunsaturated fatty acids (n-3 PUFAs) or polyphenols, can alter brain function and improve brain health through multiple pathways. NPs are first orally ingested and once in the gut, they are transformed into a wide diversity of metabolites, which can cross the intestinal epithelial barrier to reach the circulation. The metabolites generated by the gut microbiota can then undergo first-pass metabolism in the liver and phase II enzymatic conversion, such as glucuronidation and sulfation. Once in the bloodstream, NPs and gut/liver-generated metabolites can have direct effects (1) on the central nervous system (CNS) for the small subset of compounds crossing the blood–brain barrier (BBB) in sufficient quantity. Alternatively, other can have indirect effects (2) on BBB targets in brain capillary endothelial cells (BCECs) by modulating cell-signaling processes, or by balancing the influx and/or efflux mechanism under the control of several transporters (e.g., the receptor for advanced glycation end products/RAGE, low-density lipoprotein receptor-related protein 1/LRP1 and various ATP-binding cassette transporters/ABC). In addition, (3) NPs and their metabolites can improve cerebrovascular condition by enhancing cerebral blood flow, glucose uptake and/or brain oxygenation, which are critical for many CNS diseases. Further outside of the brain, circulating NPs and metabolites may impact (4) key organs regulating peripheral metabolism, to enhance the metabolic determinants, such as glucose, insulin and several metabolic hormones that might exert long-term therapeutic effects on the brain. Finally, (5) NPs can interact with the brain via the gut–microbiota–brain axis through multiple mechanisms. In part through its effect on NP metabolism, the gut microbiota can generate CNS-acting compounds in the systemic circulation, while the enteric nervous system (ENS) is connected with the CNS through the vagus nerve. Polyphenols are predominantly metabolized in the gut and the liver, thereby generating metabolites that can act on the brain through these 5 pathways. However, low brain bioavailability precludes most polyphenols to run through pathway 1. In contrast, n-3 PUFAs are more likely to act directly in the brain (pathway 1), but can also engage pathways 2, 3, 4 and 5.

If polyphenolic compounds and their metabolites act in the periphery without reaching sufficient concentrations in the cerebral tissue, it is logical to assume they will not trigger molecular mechanisms classically associated with disease modification and neuroprotection. However, the body is not just an inert receptacle for the brain. All spheres of brain integrity and function rely on constant communication with organs in the periphery. Duly messengers include hormones and other circulating compounds that do not necessarily cross the BBB. The brain also requires nutrients and sources of energy that can only come from the periphery (Figure 1).

Polyphenols are a large family of compounds that, together with their metabolites, are expected to exert a pleiotropic action on the body [145]. The possibility that they impact brain functions, including complex ones like cognition, without reaching sufficient concentrations in the CNS must be considered. Although many studies have reported changes in brain molecular endpoints after administration of polyphenols, no firm mechanism has been pinpointed in a replicable fashion [79,81,109,118,143,146,147,148]. Reports in animal models of AD or PD have shown CNS-related beneficial effects without detecting a specific brain alteration, consistent with mechanisms located outside of the brain [79,81,88,148,149,150,151].

Maintenance of cardiovascular health and, more specifically at the level of the cerebrovascular network, is essential to optimal brain function, given the reliance of the CNS on oxygen, glucose and other bloodborne nutrients. It is becoming increasing clear that a tighter control of cardiovascular risk factor decreases the risk of dementia [152,153,154]. Therefore, the reported effect of polyphenols, such as cocoa flavonoids, on blood pressure provides a compelling example of such a mechanism not involving a direct CNS action [76,81,82,95,155,156]. Regulation of cerebral perfusion is another road by which the periphery is essential for brain function. Reduced cerebral blood flow is one of the common early features of AD [157,158]. Vascular changes leading to enhanced blood flow have received significant attention as mechanisms explaining brain health impact of polyphenols [95,109,159]. Polyphenols have been linked to an enhancement of cerebral blood flow and brain oxygenation in clinical trials [81,160,161,162]. Again, such vascular effects do not require entry of polyphenols into the CNS.

Numerous studies in animal models or clinical trials have shown effects of polyphenols on metabolic determinants. Attenuation of postprandial hyperglycemia, notably through inhibition of α-amylases and α-glucosidases, as well as improvement of insulin sensitivity, have been reported [156,163,164,165,166,167,168]. This is important because metabolic defects are associated with brain diseases, particularly with AD. Type 2 diabetes (T2D), a condition characterized by impaired insulin response, is now recognized as an important risk factor for AD [169,170,171,172,173,174]. Induction of metabolic defects in the periphery of animal models of AD has been shown to aggravate brain Aβ load and, in some studies, tau pathology as well [175,176,177,178,179,180,181]. On the other hand, genetic induction of AD neuropathology leads to signs of metabolic failure in the periphery, such as glucose intolerance [182,183,184], unveiling a self-amplifying loop between T2D and AD. Therefore, multiple drugs used to treat diabetes are the subject of preclinical and clinical studies in AD such as insulin, metformin and more recently, analogues of glucagon-like-peptide 1 (GLP-1) [185]. Metabolic disorders are also associated with other CNS disorders such as schizophrenia [186,187] or PD [188,189,190] and/or their symptomatic treatment. Hence, in light of these data, a natural product that improves metabolic determinants could also be expected to exert a therapeutic effect on the brain.

The BBB itself is not just a physical obstacle between the blood and the brain, but rather a living dynamic multicellular complex, actively involved in brain homeostasis and controlling all exchanges between the brain and the periphery [191,192]. It provides a surface of 20 m^2^ for such interaction in the human brain, meaning that virtually every neuron has access to a blood microvessel in its vicinity [193]. Brain capillary endothelial cells (BCECs) are the main structural and dynamic components of the BBB, supported by pericytes and a network of astrocytes [179,192,194,195]. BCECs express numerous influx and efflux transport systems that adjust the concentrations of endogenous molecules on both sides of the BBB [179,192,195,196]. Nutrients such as glucose or amino acids cross the BBB through well-characterized transporters, but for many other bloodborne compounds, such as insulin and transferrin, their actual capacities to entirely reach the brain parenchyma remain controversial [179,197,198,199,200,201]. However, these circulating molecules may act on BBB-associated cells, by modulating cell-signaling processes or transporter expression. Therefore, given the key role played by the BBB, it is becoming clear that drugs, including phytochemicals, may influence brain function by targeting the BBB itself.

AD is a prominent example of a brain disease in which BBB transport systems play a central role, at least in certain aspects of its pathophysiology. It is increasingly recognized that cells forming the BBB can generate Aβ, but more importantly regulate its clearance out of the brain to the blood [192,202]. Data indicate that brain-to-blood Aβ clearance is impaired in AD, contributing the accumulation of plaques and other Aβ species in cerebral tissues [192,203,204]. Such a disequilibrium may implicate influx transporters (receptor for advanced glycation end-products/RAGE) and failing efflux transporters (low-density lipoprotein receptor-related protein 1/LRP1 and ABCB1/P-gp (ATP-binding cassette transporters B1/P-glycoprotein) [192,202,205,206,207,208] (Figure 1). Besides cerebral amyloid angiopathy (CAA), the BBB of AD subjects is characterized by a loss of P-gp (efflux) and neprilysin (degradation enzyme), as well as an increase in amyloid precursor protein (APP) and β-secretase (key enzyme of the amyloidogenic pathway), all in close association with ante mortem cognitive decline [202]. In contrast, other transporters like the transferrin receptor (TfR) remain unaffected in AD [209]. While no massive alteration of endothelial cells was observed [202,210], a loss of mural cells (pericytes and smooth muscle cells) has been described in AD, associated with higher vascular Aβ40 content as well as cognitive performance [211,212,213]. The overarching idea stemming from these data is that targeting production and clearance mechanisms located in the BBB can have an impact on brain proteinopathies. The possibility that polyphenol-like compounds or other natural products could act on the CNS indirectly through an effect on the BBB remains a whole promising new area to be explored.

There have been very limited reports on polyphenols exerting an effect on the BBB per se, except perhaps in vitro evidence of a modulation of the activity of ABC transporters [113], which are involved in Aβ40 clearance [214,215]. Indeed, flavonoids may act as substrates and/or modulators of membrane-bound transport proteins (such as ABC transporters), thereby possibly altering the bioavailability of drugs, toxins and bioactive food molecules, including other phytochemicals [113,216]. The exact mechanisms by which circulating phenolic metabolites interact with the BBB therefore needs to be further investigated.

The gut–microbiota–brain axis represents another intriguing pathway by which orally administered natural products may alter brain activity. Polyphenols are well known to be highly metabolized by gut microbiota, generating an array of bioactive metabolites [115,217]. Conversely, polyphenolic compounds exert prebiotic effects on the gut microflora, modifying bacterial composition and function [115,218,219,220]. Compelling evidence suggests that metabolic effects of polyphenol are mediated by gut-microbiota-dependent mechanisms [115,219,221,222,223]. Impairments in immunological and metabolic processes, mediated by an altered gut microbiome, contribute to the onset and progression of cognitive disorders [220]. Recent studies have pinpointed that gastrointestinal dysfunction and the resulting alteration in gut-microbiota composition are associated with the development of CNS diseases, for which polyphenols could be used as therapeutics through prebiotic activities [224,225]. Of note, the gut is innervated by the enteric nervous system (ENS), which is connected to the brain through vagal pathways, thereby providing a direct neural link between diet, the gastrointestinal tract, its microbiota and the brain [226,227]. The question of whether interactions between ingested polyphenols and the gut ultimately exert an effect on cerebral activity is beginning to be explored [122], but the answer may involve all mechanisms discussed above.

Most mechanisms requiring direct target engagement or physicochemical effects of polyphenolic compounds can probably be ruled out due to their low CNS concentrations. It used to be assumed that polyphenols exerted their action through an antioxidant action, but this has been challenged, as very high concentration at the target sites would be necessary [75,112,122,228]. Obviously, minimal concentrations required to exert such an antioxidant effect are very unlikely to be reached in vivo in the brain.

Finally, it can be expected that the extra-CNS action of natural products will be slower than when resulting from a direct interaction with CNS targets. Most CNS drugs exert their effect within minutes or hours. However, it is well known that some therapeutic agents, such as antidepressants or antiepileptics, show most of their benefits only after weeks of daily administration. It is also increasingly recognized that neurodegenerative diseases should be treated as early as possible, using primary and secondary prevention approaches, years before the occurrence of symptoms. Then, a relatively gentle and sustained intervention extended over several years may have a reasonable chance to exercise its neuroprotective effect without using a chemical crossing the BBB. Migraine is another prevalent condition that can be prevented by chronic intake of drugs. Overall, these types of indications, requiring a slow response over a long period of time may have better chance to benefit from the extra-CNS effect of natural products.

## 7. On the Importance of Investigating Bioavailability Early in CNS Drug Development

Animal models are not like little humans or little patients, and their value to predict clinical efficacy is sometimes overestimated. Although there are many examples of translational successes in specific CNS disorders, such as for epilepsy, the reliance on animal models has turned out to be less effective in drug development for neurodegenerative diseases [229,230,231,232]. Unfortunately, most animal models recapitulate only a fraction of the constellation of etiophysiopathological events of prevalent complex diseases. Higher cognitive function and complex symptoms, such as anxiety and mood disorders, cannot be truly deduced from simple animal-behavior paradigms, which are nevertheless widely accepted as gold standards in preclinical studies.

In contrast, data generated in animal models may prove particularly valuable in predicting ADME and PK parameters, including CNS penetration in humans [7]. While the enzymatic machinery involved in metabolism and transporters (ie P-gp substrates) can differ between rodents and humans, key features are usually similar. Therefore, they can be extremely useful for bioavailability studies, a purpose for which they are probably underused, at least in the academic setting, probably due to limited available funding. For example, the physicochemical characteristics underlying the capacity of a drug to cross the BBB remain the same in the mouse or in primates [7,66,233]. Still, many drugs for CNS applications reach phase III along with relatively limited preclinical evaluation of PK and bioavailability [2,3,234]. Body distribution favoring interaction with the pathogen in the infected organs has been central in the selection of the right antibiotics [235]. The same is true for CNS use [2,3,234]. Therefore, it is suggested that the documentation of ADME and PK and CNS distribution should deserve as much investment as efficacy studies in preclinical phases. When studying complex natural products, interactions with other components within dietary sources have to be taken into account as well in animal studies.

## 8. General Conclusions

In this review, we have attempted to summarize the scientific evidence supporting the effects on brain cognition of two classes of natural products, n-3 PUFAs and polyphenols. Despite numerous gaps in our knowledge and probable publication biases, the sum of evidence accumulated so far is remarkable and difficult to ignore. While the brain accretion of n-3 PUFAs following oral intake is well documented, the CNS effects of polyphenols are harder to reconcile with their low brain bioavailability. Whereas classical neuropharmacology teaches us that reaching minimal concentrations at the target site is a condition sine qua non for efficacy, it ignores the fact that the brain is not isolated and that it is modulated by events in the periphery. We have provided a list of mechanisms outside of the CNS per se by which polyphenols and other natural products can effectively alter brain function and health, notably through the brain vasculature, the BBB itself or the gut–brain axis (Figure 1). These extra-CNS effects might be particularly suitable for long-term preventive effects against slow progressive diseases, such as AD.

Important limitations and their impact on future perspectives remain to be considered. It is critical to consider that natural products are usually more complex from a chemical point of view that the typical synthetic compound. They also cannot be dissociated from their dietary sources (e.g., fruits and vegetable for polyphenols and fish for n-3 PUFAs), which must be taken into account when interpreting associative data, and in the design of future intervention studies. In addition, utilizing preclinical data for human health research questions remains a challenge. Animal studies can provide extremely useful information, particularly when measuring bioavailability or BBB transport, but can be flawed due to methodological issues.

We end with a call for bioavailability and PK studies early in drug development, as they provide decisive information on the potential for translation into clinics. Investigations of biodistribution (PK studies) and mechanisms (pharmacodynamics studies) should go hand in hand in the earliest phases of studies on any natural product.

## Data Availability

No new data were created or analyzed in this study. Data sharing is not applicable to this article.

## Abbreviations

ABCB1/P-gp	ATP-binding cassette transporters B1/P-glycoprotein
ACSL6	Acyl-CoA synthetase long chain family member 6
AD	Alzheimer’s disease
ADAS-Cog	AD Assessment Scale–Cognitive Subscale
ADME	absorption, distribution, metabolism, excretion
ApoE4	apolipoprotein ε4
APP	amyloïd precursor protein
ARA	arachidonic acid
AUC	area under the curve
Aβ	β-amyloid
BBB	blood–brain barrier
BCEC	brain capillary endothelial cells
CAA	cerebral amyloid angiopathy
CAIDE	Cardiovascular Risk Factors, Aging, and Incidence of Dementia
CNS	central nervous system
CSF	cerebrospinal fluid
DHA	docosahexaenoic acid
EGCG	epigallocatechin gallate
ENS	enteric nervous system
EPA	eicosapentaenoic acid
FA	fatty acid
FAPB5	fatty acid binding protein 5
GLP-1	glucagon-like-peptide 1
LRP1	low-density lipoprotein receptor-related protein 1
MEC	minimum effective concentration
MMSE	Mini-Mental State Examination
n-3 PUFAs	omega-3 polyunsaturated fatty acids
NPs	natural products
PD	Parkinson’s disease
PK	pharmacokinetic
RAGE	receptor for advanced glycation end-products
RCT	randomized controlled trial
T2D	type 2 diabetes
TfR	transferrin receptor.
